# Scanning Electron Microscopy Analysis of the Intratubular Radicular Dentin Penetration of Calcium Hydroxide, Triple Antibiotic Paste, and Nitrofurantoin

**DOI:** 10.3390/jpm13111554

**Published:** 2023-10-30

**Authors:** Unmesh Khanvilkar, Sanika Pawar, Siddhesh Bandekar, Vaishnavi Dhok, Suraj Arora, Ajinkya M. Pawar, Francesco Pagnoni, Rodolfo Reda, Luca Testarelli

**Affiliations:** 1 Department of Conservative Dentistry and Endodontics, Yogita Dental College and Hospital, Khed, Ratnagiri 415709, Maharashtra, India; unmesh22@yahoo.com (U.K.); sanikapawar37@gmail.com (S.P.); siddheshbandekar1987@gmail.com (S.B.); vaishnavi24d@gmail.com (V.D.); 2 Department of Restorative and Dental Sciences, College of Dentistry, King Khalid University, Abha 61421, Saudi Arabia; surajarorasgrd@yahoo.co.in; 3 Department of Conservative Dentistry and Enododntics, Nair Hospital Dental College, Mumbai 400008, Maharashtra, India; 4 Department of Oral and Maxillofacial Sciences, Sapienza University of Rome, 00161 Rome, Italy; pagnoni.1847035@studenti.uniroma1.it (F.P.); luca.testarelli@uniroma1.it (L.T.)

**Keywords:** intracanal medication, radicular dentine penetration, calcium hydroxide, triple antibiotic paste, nitrofurantoin, scanning electron microscopy

## Abstract

The aim of this study is to assess and analyze the intratubular penetration of the intracanal medications nitrofurantoin (Nit), triple antibiotic paste (TAP), and calcium hydroxide (CH). Sixty freshly extracted single-rooted teeth were acquired and decoronated to a standard length of 15 mm. To prepare specimens up to size F3, rotary ProTaper instrumentation was employed. The prepared teeth were divided into three groups, each of which received one of the tested intracanal medicaments: Group I (calcium hydroxide), Group II (triple antibiotic paste), and Group III (nitrofurantoin). Using a size #30 Lentulo spiral, a freshly prepared therapeutic paste was placed into the canals, and the intracanal medicaments were allowed to set in the incubator at 100% humidity. The samples were subsequently sliced perpendicularly to their long axis using a precision saw and assessed under a scanning electron microscope to assess the depth of penetration of intracanal medicaments at the coronal, middle, and apical portions of the root canal dentin. The data were analyzed using one-way ANOVA and Tukey’s post hoc test. The statistical analysis revealed a significant difference between the experimental groups in the quantity and depth of sealer penetration (*p* < 0.05). In particular, as compared to the Nit group, both the CH and TAP groups had significantly smaller penetration areas (*p* < 0.05). In conclusion, this ongoing investigation indicates that nitrofurantoin penetrated dentinal tubules better than calcium hydroxide or triple antibiotic paste.

## 1. Introduction

Intracanal drugs play an important role in battling against pathogens in the root canal. These drugs are crucial in modern endodontic therapy, where the focus is typically placed in shaping and completely cleaning the root canal, which might take precedence over the selection of intracanal disinfectants. When endodontic therapy cannot be completed in a single visit, intracanal medicaments are usually indicated as a crucial technique to address the continuous problem of tenacious intracanal bacteria, which have been proven to grow between treatment sessions [1]. The fundamental goal of endodontic intracanal therapy is to effectively remove bacterial flora within the root canal and, in some situations, to improve bacterial growth control. Interappointment antimicrobial treatment is critical to this procedure because it efficiently inhibits bacterial growth, removes surviving microorganisms, and prevents pathogen ingress via any inadequately sealed or leaking restorations [2]. This highlights the importance of intracanal drugs in not only eradicating infections that remain but also reduces the likelihood of reinfection. Thus, the need of meticulousness in endodontic operations is highlighted, providing thorough cleaning and excellent results [3,4].

One of the key objectives of endodontic therapy is to successfully limit pathogen development and proliferation inside the complex network of the root canal space. Once a microbial infection has taken hold, these resistant bacteria can invade the dentin isthmus, apical delta, lateral canal, and primary root canal. This broad penetration, on the other hand, poses a substantial barrier for standard biomechanical preparation approaches, which may not always be successful in completely eradicating all harmful microorganisms. As a result, intracanal medicaments must penetrate deeply into the intricate network of dentinal tubules to effectively address intracanal bacteria, avoiding the creation of chronic and recurring infections. By guaranteeing that these medications reach the dentin’s deepest depths, the therapy can seek to not only remove existing infections but also to develop a strong defense against the reemergence of pathogenic bacteria. As a result, complete pathogen elimination and the avoidance of chronic infections inside the root canal system are emphasized as critical goals in effective endodontic therapy [4].

Bacteria have an important role in the genesis and progression of pulpal and periapical diseases. At depths ranging from 200 to 1500 μm [5,6], they enter the dentinal tubules via the root canal system. Dentinal tubule bacteria limit the efficacy of systemic antibiotics as well as the body’s own immune systems. Due to its anatomical intricacy, the root canal system has restricted access to instruments and irrigants, making complete bacterial eradication difficult to attain. As a consequence, antibacterial intracanal medications are strongly advised for full sanitization. Traditional antibiotics are efficient against pathogens; however, they are unable to reach the dentinal tubules, where they are required [5,7].

Calcium hydroxide (CH) is a prevalent intracanal medication that may eradicate microorganisms that dissolve organic tissue, and relieve inflammation. This material has a high pH, ranging from 12.5 to 12.8. The dissociation of calcium (Ca^2+^) and hydroxyl (OH^−^) ions contributes greatly to CH’s capabilities, including its expected antibacterial effect, tissue dissolution, tooth resorption prevention, and hard tissue regeneration encouragement [8,9,10]. The porous nature of dentine allows for the diffusion of isolated particles via dentinal tubules. Solute features such as dentinal tubule architecture, density, width and length, and molecular weight all influence dentine permeability. Calcium hydroxide increases the pH inside the dentinal tubules, allowing bacteria to be annihilated and antimicrobial properties to be created [11,12].

Endodontic procedures and therapeutic techniques, such as pulp neo-vascularization therapy, frequently need the use of antibiotic pastes as a vital component of the treatment protocol. The triple antibiotic paste (TAP) has emerged as a remarkable choice among these pastes. TAP is differentiated by its strong antibacterial properties, making it a formidable candidate for the management of difficult endodontic circumstances. In addition, TAP has received praise for its biocompatibility, which aligns it with the criteria of modern endodontic therapy, which prioritizes the preservation of both the patient’s health and the integrity of the treated tooth. Hoshino et al. [12] implemented it in their study, and it contains ciprofloxacin, metronidazole, and minocycline. It was verified in a clinical study and case series that employing TAP as an antibacterial dressing during conventional root canal therapy was effective in resolving significant cyst-like periradicular lesions without the use of surgery. Furthermore, another investigation found that TAP alleviated clinical symptoms such as edema, sinus tracts, induced or spontaneous dull pain, and discomfort on biting in primary teeth with peri radicular lesions [13,14]. Despite its advantages, new research indicates that TAP should be adopted with caution. First, because of the possibility of tooth discoloration due to TAP, minocycline should only be used in the root canal. Second, at high dosages, TAP may be cytotoxic [15].

Nitrofurantoin (Nit) is a nitrofuran compound which has broader antibacterial action against both Gram-positive and Gram-negative bacteria. It is an effectively known oral antibiotic that is used to treat urinary tract infections (UTIs) as well as infections caused by multidrug-resistant bacteria. Notably, studies have shown that Nit is particularly efficient against E. faecalis, even when other antibiotics’ effectiveness is decreased [2,16].

Alrahman et al. [2] evaluated nitrofurantoin as a speculative intracanal medicament in endodontics and noticed that at a concentration of 25 mg/mL, Nit paste productively eliminated EF when used as an intracanal medicament. In contrast, not enough research has been conducted to investigate the depth of nitrofurantoin paste penetration into dentinal tubules. As a consequence, the intention of this investigation is to analyze and investigate the intratubular penetration of calcium hydroxide, triple antibiotic paste, and nitrofurantoin paste using scanning electron microscopy. We anticipate that there will be no substantial difference in intratubular penetration between the three medications. 

## 2. Materials and Methods

A combined total of 60 newly removed 60 single-rooted single-canaled mandibular premolar teeth were cleaned with an ultrasonic scaler of calculus and other debris before being kept in saline and awaiting usage. A power analysis was performed with G*Power version 3.0.1 (Franz Faul Universität, Kiel, Germany). A total minimum calculated sample size of 60 samples (20 samples per group; total of 3 groups) would require to yield 80% power to detect significant differences, with an effect size of 0.42 [17] and a significance level at 0.05.

The teeth with an apical diameter preoperatively coinciding with #20 k-file were only selected for standardization of instrumentation till #30 apical diameter. A water-cooled double-sided disc moving at low speed in a handpiece was implemented to decoronate teeth at the cementum–enamel proximity, leading to a uniform root length of 15 mm. By introducing a K-file size #10 (Mani), the canal patency was validated. The working length was identified by inserting a K-file size #15 into the apical foramen and subtracting 1 mm from this measurement. The teeth were then all instrumented applying the Pro Taper universal rotary system (Dentsply) and prepared to F3 apical diameter. The root canals were then irrigated with 3 mL of 5.25% sodium hypochlorite in between each file while instrumenting them with a 27-gauge needle, which was 1 mm shorter than the working length, followed by 3 mL of saline solution and 3 mL of 17% EDTA for 1 min to eliminate the smear layer. Finally, the canals were rinsed with 3 mL of saline solution and dried with sterile absorbent paper points (Dentsply) before being categorized into three groups (Table 1). 

### 2.1. Preparation of Medicaments

#### 2.1.1. Calcium Hydroxide

The calcium hydroxide powder was blended in a 1:1 ratio with distilled water using a glass slab and a stainless-steel spatula.

#### 2.1.2. Triple Antibiotic

In search of the appropriate TAP paste concentration for usage in clinical settings (1 g/mL), the compounding of United States Pharmacopeia (USP) grade antibiotic powders, particularly metronidazole, ciprofloxacin, and minocycline, was performed with utmost accuracy and care. This was achieved by mixing 1 g of the aforementioned granules with 1 milliliter of pure sterile water [18].

#### 2.1.3. Nitrofurantoin

To attain the desired consistency, a thick paste-like mixture was created by combining a crushed tablet of Niftas (Intas Pharmaceuticals Ltd., Majhitar, Sikkim, India) to obtain NIT powder using distilled water (DW). To improve its characteristics, Methylcellulose (MC) powder from Akshar Chemicals Ltd. Dahej, Gujarat, India, was incorporated into the NIT solution. To produce 100 mg/mL of NIT paste, 100 mg NIT + 1 mL DW + 80 mg MC were combined [19].

By employing a size #30 Lentulo spiral anchored to a contra-angle handpiece and micromotor, the prepared pastes were inserted into the root canals. To prevent leaking, the coronal openings of the root canals were stuffed with temporary filling materials (3M ESPE, Seefeld, Germany) and sealed with compact cotton pellets. Following that, the specimens were kept in an incubator for three weeks at a temperature of 37 °C and a humidity of 100%.

Each of the samples was sectioned perpendicularly to its long axis using a precision saw at a moderate speed while being cooled by water to assess the penetration of medications. Each tooth was cut into three slices with apical, middle, and coronal depths of 4, 7, and 10 mm and a thickness of around 2 mm. Each specimen was placed within a circle-shaped self-curing acrylic resin mold, and a scanning electron microscope (Hitachi SU3500 SEM, Tokyo, Japan) was used to determine the extent of the penetration of the medications. Using a calibrated measuring instrument built into the microscope, measurements for the depth of penetration of the intracanal medications were recorded in the coronal, middle, and apical thirds of the root canal at a magnification of 200×.

### 2.2. Statistical Analysis

The data were input into Microsoft Excel 2010. For each group, the descriptive data for penetrations were presented as means and standard deviations (SDs). Analysis of variance (ANOVA) was used to compare the penetrations of the three groups, and Tukey’s post hoc analysis was used to compare groups on a pair-wise basis. When the test’s ‘*p*’ value was 0.05 or below, all of the results were deemed statistically significant. Version 19 of SPSS (Statistical Package for Social Sciences) was the program utilized.

## 3. Results

The results showed a statistically significant disparity in penetration between the three groups at the overall level, with *p* < 0.001 * and F = 98.724. Table 2 shows the overall findings of an examination of intratubular penetration among the three groups (calcium hydroxide, triple antibiotic paste, and nitrofurantoin) using ANOVA and Tukey’s post hoc test. Nitrofurantoin consistently had the greatest depth of penetration (mean: 991.4510 μm), with significant differences when compared to both calcium hydroxide (mean difference: −473.0560) and triple antibiotic paste (mean difference: −308.9595), as demonstrated by *p*-values of 0.001. 

**Figure 1:**
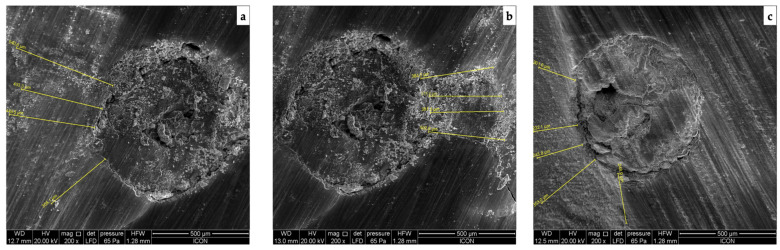
SEM micrographs of the penetration into the radicular dentine in group with calcium hydroxide as intracanal medicament at (**a**) coronal, (**b**) middle, and (**c**) apical third.

**Figure 2:**
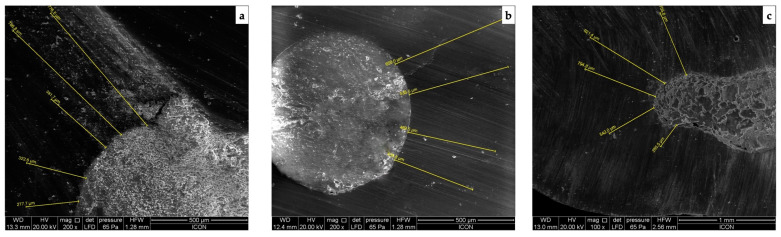
SEM micrographs of the penetration into the radicular dentine in group with triple antibiotic paste as intracanal medicament at (**a**) coronal, (**b**) middle, and (**c**) apical third.

**Figure 3:**
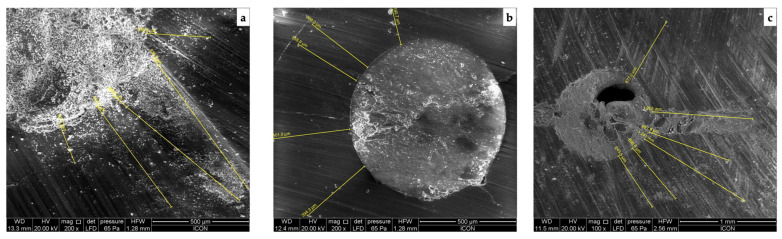
SEM micrographs of the penetration into the radicular dentine in group with nitrofurantoin paste as intracanal medicament at (**a**) coronal, (**b**) middle, and (**c**) apical third.

Figure 1 (calcium hydroxide), Figure 2 (triple antibiotic paste), and Figure 3 (nitrofurantoin) show each group’s representative depictions.

Table 3 compares intratubular penetrations amongst the three research groups, especially at the coronal level. The mean penetration depths for each group are shown, along with their standard deviations (SDs). The mean penetration of calcium hydroxide (Group I) is 518.3950 μm; however, triple antibiotic paste (Group II) and nitrofurantoin (Group III) have higher mean penetrations at 950.4915 μm and 991.4510 μm, respectively. Findings from Tukey’s post hoc tests show substantial differences in penetration depth between the three groups, as demonstrated by *p*-values of 0.001, emphasizing the statistical significance of the discrepancies.

Table 4 outlines the analysis of intratubular penetrations at the middle level among the three groups (calcium hydroxide, triple antibiotic paste, and nitrofurantoin) using ANOVA and Tukey’s post hoc test. Nitrofurantoin had the deepest penetration (mean: 624.5085 μm) at this intermediate level, with significant differences compared to both calcium hydroxide (mean difference: −387.6195) and triple antibiotic paste (mean difference: −249.8650), as demonstrated by *p*-values of 0.001.

Table 5 highlights the ANOVA and Tukey’s post hoc test results for intratubular penetrations at the apical level in the three groups (calcium hydroxide, triple antibiotic paste, and nitrofurantoin). Nitrofurantoin continued to have the greatest apical penetration (mean: 868.6655 μm), with significant differences when compared to calcium hydroxide (mean difference: −426.2805) and triple antibiotic paste (mean difference: −293.308), as evidenced by *p*-values of 0.001.

## 4. Discussion

The eradication of infections and their metabolites is the major goal of endodontic therapy. To accomplish this, a mix of instruments, irrigation solutions, and medications should be employed [20]. An intracanal medicament has to penetrate deeply and densely into the dentinal tubules in order to have advantageous antibacterial and obstructive consequences against reinfection. The surface resistance of an intracanal medicament influences its capacity to permeate the dentinal tubules, with low surface tension allowing improved penetration into inaccessible places. Furthermore, dentinal tubule architecture, density, diameter, length, and solute properties (such as size and charge) all have an effect on dentinal permeability. Variations in dentin’s tube density and size have also been documented in the radicular dentin, notably in the apical area of permanent tooth roots [21]. Mjor et al. [22] discovered that dentinal tubules were uneven in direction and density at the apical region of permanent tooth roots.

Dentinal tubules have been estimated to have a diameter of 2 to 4 µm. The scanning electron microscope has already been shown to be an excellent instrument for measuring the extent of material penetration into these complex dentinal tubules in prior research investigations. This is partly due to its excellent ability to offer complete and realistic visual depictions of dentinal tubules and their contents, allowing researchers to investigate the complexities of these microstructures with great accuracy. Nonetheless, it is critical to recognize that using this powerful imaging technology comes with its own set of obstacles. Conducting systematic analysis at lower magnification levels, in particular, can be intrinsically challenging, and the sample preparation procedure has the potential to contribute unwanted outcomes that may impair the accuracy of the outcomes. As a result, while the scanning electron microscope provides a significant glimpse into what is occurring in dentinal tubules, researchers must take caution in both its use and the related preparations to ensure the validity of their results [23].

The smear layer, material chemical and physical attributes, and tooth morphology all have an effect on material penetration into dentinal tubules [24]. The use of EDTA and NaOCl, together, has been found to effectively eliminate the smear layer formed during endodontic treatment. EDTA works on the smear layer’s inorganic components, demineralizing peritubular and intertubular dentin and releasing collagen. Following that, NaOCl acts to dissolve the collagen, further opening and exposing the dentinal tubules [25]. The smear layer removal procedure exposes dentinal tubule apertures, allowing material to penetrate the tubules at different depths [26]. As a result, in this investigation, NaOCl and EDTA were utilized in all groups to remove the smear layer. The medications allow for greater penetration into dentinal tubules.

Nitrofurantoin is a powerful antibacterial agent with an extended track record of success in treating lower urinary tract infections (UTIs). Multiple trials have also shown that it is effective against Enterococcus faecalis [27]. Alrahman et al. [2] found that nitrofurantoin paste, at a dose of 25 mg/mL, is effective in completely eradicating E. faecalis when used as an intracanal medicament. They also investigated the biocompatibility of nitrofurantoin as an exploratory intracanal medicament in endodontic treatment. They additionally confirmed that the two nitrofurantoin paste concentrations (12.5 mg/mL and 25 mg/mL) used in the investigation were biocompatible with rat subcutaneous connective tissue [2].

To the best of our knowledge, no data on the depth of penetration of nitrofurantoin paste into the complicated network of dentinal tubules has been published in the scientific literature. The primary goal of this study was to investigate the intratubular penetration of three different intracanal medications: nitrofurantoin, triple antibiotic paste, and calcium hydroxide. The use of a scanning electron microscope substantially aided our investigation, allowing for a detailed assessment of the amount to which these medications entered the complicated network of dentinal tubules. We commenced our investigation based on our initial null hypothesis, which stated that there would be no substantial difference in the intratubular penetrations among these medications. Interestingly, our findings deviated significantly from this notion. They determined that the nitrofurantoin group penetrated the dentinal tubules much deeper than the triple antibiotic paste and calcium hydroxide groups. As a result, we were forced to reject the null hypothesis, indicating that the intratubular penetrations of these drugs differed considerably.

Each endodontic intracanal medicament has the capacity to affect the physical and mechanical characteristics of the radicular dentin. The efficacy of intracanal medicine was much stronger in the coronal third, less effective in the middle third, and least effective in the apical third (according to Abdulrahman et al. [28]), with a very significant difference between these locations. The difference in tube density on the root dentin surface from the cervical to apical areas reflects this disparity. Furthermore, there was an inverse relationship between dentine microhardness and tubular density, as Pashley et al. [29] reported that dentine microhardness decreases as tubular density increases. As an effect, there is a strong positive relationship between phosphate/amide I ratios and dentine microhardness. Furthermore, this is due to a decrease in the quantity of the calcified matrix between tubules as tubule density increases, which is also related with a decrease in the amount of intertubular dentin, leading in lower phosphate/amide I ratios. As an outcome, a diminution in microhardness might raise the solubility and permeability of the root canal dentine, lessening the sealing ability and adhesion of dental materials to the dentine and facilitating bacterial invasion coronal leakage [28].

Given that root canal infections are so complicated, antibiotic combinations may be required to treat the diverse bacterial flora involved with such illnesses. In accordance with a particular study, the most effective component of the triple antibiotic paste against E. faecalis is minocycline. Minocycline, a semisynthetic tetracycline derivative, is effective against both Gram-positive and Gram-negative bacteria [23].

In a retrospective study, Bose et al. [30] suggested that the application of both calcium hydroxide (Ca(OH)_2_) and a triple antibiotic paste can possibly promote the ongoing development of the pulp–dentin complex in cases involving immature necrotic teeth, pointing to antibacterial properties as a contributing factor [29,31,32]. Additionally, Adl et al. [21] investigated the antibacterial activity of calcium hydroxide and a triple antibiotic paste against Enterococcus faecalis, a notably robust bacterium in endodontic infections. Their findings not only disclosed that the effectiveness of the triple antibiotic paste outperformed calcium hydroxide pastes as an intracanal medicament for treating Faecalis-related infections, but also identified minocycline as the most potent component within the triple antibiotic paste. These findings shed light on how to effectively address endodontic difficulties caused by chronic and resistant bacteria.

Calcium hydroxide (Ca(OH)_2_) is one of the most commonly employed intracanal medicaments in the field of endodontics. Notably, both short-term and long-term applications of Ca(OH)_2_ within the root canal have revealed possible negative aspects since they might have an unfavorable effect on the chemomechanical properties of the root dentin. This impact might be linked to the high pH values associated with calcium hydroxide, which can harm the surface collagen of the root dentin. While pure Ca(OH)_2_ may not be considered particularly effective as a broad-spectrum root canal medicament, various research studies have offered evidence demonstrating that it has limited efficacy against rigid, resistant pathogens that occur frequently within the root canal system [9]. This, in turn, contributed to the continued search for more effective ways and agents to tackle these infections. 

When calcium hydroxide particles reach the complicated network of dentinal tubules, they come into direct contact with the microorganisms that thrive there, acting as a direct source of dissociated calcium ions. These calcium hydroxide particles dissolve in water over time, resulting in the formation of hydroxide ions (OH^−^). This dynamic mechanism is critical in keeping a constant pH level inside the root canal environment. As a result, the antibacterial effect of calcium hydroxide is greatly enhanced, while the potential for the pH to drop is minimized by the dentin’s own buffering characteristics. These diverse activities add to the complicated interplay of elements that characterizes calcium hydroxide’s role as an intracanal medicament in endodontic therapy [32].

The nitrofurantoin group had a considerably better depth of penetration in the apical third than the triple antibiotic paste and calcium hydroxide groups. This might be due to a variety of causes, including the miniscule size of the nitrofurantoin particles, which allows for deep penetration into the dentinal tubules to eliminate pathogens that have made their way there. Furthermore, the presence of characteristics such as uneven secondary dentin apical surfaces, auxiliary root canals, and the resorption or healing of resorption sites in the apical area may contribute to nitrofurantoin’s improved penetration. The triple antibiotic paste had the highest mean intratubular penetration in this study, whereas calcium hydroxide had the lowest [2].

TAP is not adequately eliminated from the root canal system, according to Birkhoff et al. [33], regardless of the irrigation strategy utilized, with more than 80% of TAP persisting in the root canal system. On the other hand, CH was efficiently eliminated, and residues were considerably decreased. TAP was shown to diffuse circumferentially up to 350 µm into the dentin in the same research. These findings were attributed by the researchers to TAP’s penetration and binding to dentin. The size, shape, and orientation of calcium particles, according to Komabayashi et al. [34], can regulate the depth of calcium hydroxide’s penetration into the dentinal tubules. The greater the particle size, the more rectangular the particle form, and hence more ideal for deep penetration.

Nitrofurantoin and antibiotic combinations, particularly minocycline, have the potential for prolonged persistence inside the dentin. Although the conceivable negative effects of the other intracanal medications are unknown, these residual intracanal medications within the dentin may create drug-resistant microorganisms or result in cytotoxic consequences. As a result, future research should be carried out to improve the removal efficiency of nitrofurantoin paste when it is utilized as an intracanal medicament.

## 5. Conclusions

In conclusion, our research found a substantial difference in penetration depth between calcium hydroxide, triple antibiotic paste, and nitrofurantoin. Nitrofurantoin paste, in particular, penetrated deeper into the dentinal tubules than both calcium hydroxide and triple antibiotic paste under the conditions of this study. Notably, all of the investigated medications showed a similar decline in penetration depth from the coronal to apical areas. These findings highlight the relevance of substance selection in maximizing treatment results and provide useful insights for clinical and research applications in dentistry and medical settings.

## Figures and Tables

**Table 1:** Experimental groups of the study.

Specimen	Groups (*n* = 20)	Intracanal Medicament
*n* = 60	Group 1	Calcium hydroxide paste (Prevest DenPro Limited, Bari Brahmana, Jammu, India)
	Group 2	Triple antibiotic paste Metronidazole (J.B. Pharmaceuticals Ltd., Ahmedabad, Gujrat, India) Ciprofloxacin (Sun Pharmaceutical India Ltd., Mumbai, India) Minocycline (Sun Pharmaceutical India Ltd., Mumbai, India)
	Group 3	Nitrofurantoin paste (Intas Pharmaceuticals Ltd., Majhitar, Sikkim, India)

**Table 2:** Comparison of penetration among three groups (μm) at overall level using ANOVA followed by Tukey’s post hoc test.

	Groups	Overall		Tukey’s Post Hoc Test			
		Mean	SD		I vs. II	I vs. III	II vs. III
	I—Calcium hydroxide	399.2230	137.96098	**Mean difference**	−144.94117	−428.98533	−308.9595
	II—Triple antibiotic	798.1642	182.56640	**Standard error**	31.05971	31.05971	31.05971
	III—Nitrofurantoin	828.2083	207.27545	**Lower bound**	−218.3538	−502.3980	−357.4568
ANOVA	**F VALUE**	98.724		**Upper bound**	−71.5285	−355.5727	−210.6315
	***p* VALUE**	0.001 *		***p* value**	0.001 *	0.001 *	0.001 *

* Statistically significant values.

**Table 3:** Comparison of penetration among three groups (μm) at coronal level using ANOVA followed by Tukey’s post hoc test.

	Groups	Coronal		Tukey’s Post Hoc Test			
		Mean	SD		I vs. II	I vs. III	II vs. III
	I—Calcium hydroxide	518.3950	67.75172	**Mean difference**	−164.0965	−473.0560	−308.9595
	II—Triple antibiotic	950.4915	123.29037	**Standard error**	32.82038	32.82038	32.82038
	III—Nitrofurantoin	991.4510	146.95458	**Lower bound**	−243.0761	−552.0356	−387.9391
ANOVA	**F VALUE**	107.121		**Upper bound**	−85.1169	−394.0764	−229.9799
	***p* VALUE**	0.001 *		***p* value**	0.001 *	0.001 *	0.001 *

* Statistically significant values.

**Table 4:** Comparison of penetration among three groups (μm) at middle level using ANOVA followed by Tukey’s post hoc test.

	Groups	Middle		Tukey’s Post Hoc Test			
		Mean	SD		I vs. II	I vs. III	II vs. III
	I—Calcium hydroxide	236.8890	51.90199	**Mean difference**	−137.7545	−387.6195	−249.8650
	II—Triple antibiotic	374.6435	110.46255	**Standard error**	32.84964	32.84964	32.84964
	III—Nitrofurantoin	624.5085	132.20125	**Lower bound**	−216.8045	−466.6695	−328.9150
ANOVA	**F VALUE**	71.559		**Upper bound**	−58.7045	−308.5695	−170.8150
	***p* VALUE**	0.001 *		***p* value**	0.001 *	0.001 *	0.001 *

* Statistically significant values.

**Table 5:** Comparison of penetration among three groups (μm) at apical level using ANOVA followed by Tukey’s post hoc test.

	Groups	Apical		Tukey’s Post Hoc Test			
		Mean	SD		I vs. II	I vs. III	II vs. III
	I—Calcium hydroxide	442.3850	84.67199	**Mean difference**	−132.9725	−426.2805	−293.308
	II—Triple antibiotic	825.3575	115.72070	**Standard error**	34.35365	34.35365	34.35365
	III—Nitrofurantoin	868.6655	144.52619	**Lower bound**	−215.6418	−508.9498	−375.9773
ANOVA	**F VALUE**	80.617		**Upper bound**	−50.3032	−343.6112	−210.6387
	***p* VALUE**	0.001 *		***p* value**	0.001 *	0.001 *	0.001 *

* Statistically significant values.

## Data Availability

The data presented in this study are available upon request from the corresponding authors.
